# Dental Caries and Its Causes

**Published:** 1873-08

**Authors:** 


### Books Reviewed.

*Dental Caries and its Causes.* An Investigation into the Influence of Fungi in the Destruction of the Teeth. By Drs. Leber and Rottenstein. Translated by Thomas H. Chandler, D. M. D. With Illustrations. Philadelphia: Lindsay & Blackiston, 1873. Buffalo: T. Butler & Son.

The authors do not pretend to present a monograph on the subject of Dental Caries, but to simply give the result of their own observations. The book consists of four sections. Part first gives a resume of the investigations made up to the present time into the Nature of Dental Caries. This gives in a brief space an account of the more important investigations which have been made, and the conclusions arrived at by different writers. Part second considers the anatomical alterations in teeth during Caries. Part three is devoted to a consideration of the progress and symptoms of Caries of the teeth, and part four to its causes. The influence of acids in producing Caries is duly considered—and the conclusions arrived at lead to some valuable therapeutical facts. The authors attach much importance to the agency of Leptothrix Buccalis, a fungus which is almost constantly found in the Buccal Cavity, unless care is taken to rinse the mouth frequently. In an examination of forty persons by Mr. Bowditch those only were found to be free who cleansed their teeth several times a day, and at least once with soap. The book is well written, and the views of the authors clearly stated. It will be read with interest by all, and is especially valuable to Dentists.

